# The Concentration of Micronutrients and Heavy Metals in Maternal Serum, Placenta, and Cord Blood: A Cross-Sectional Study in Preterm Birth

**DOI:** 10.1155/2019/5062365

**Published:** 2019-01-01

**Authors:** Rima Irwinda, Noroyono Wibowo, Atikah Sayogo Putri

**Affiliations:** Maternal Fetal Division, Department of Obstetrics and Gynecology, Faculty of Medicine, Universitas Indonesia/Cipto Mangunkusumo Hospital, Jakarta 10430, Indonesia

## Abstract

**Background:**

Preterm birth is still a global burden particularly in Indonesia. The suboptimal concentration of certain micronutrients and heavy metals is hypothesized to play a role in the mechanism of preterm birth.

**Objective:**

This study aimed to analyze the micronutrients and heavy metals concentrations between subjects with term and preterm birth.

**Design:**

A cross-sectional study was conducted during January–June 2017 in Cipto Mangunkusumo Hospital and Budi Kemuliaan Hospital, Jakarta, Indonesia. Subjects were divided into term and preterm birth groups. The measured outcomes were maternal serum, placental, and blood cord concentration of zinc, copper, iron, selenium, manganese, mercury, lead, AtRA, and 25(OH)D.

**Results:**

A total of 51 pregnant women participated in this study. Term group had higher concentration of maternal serum AtRA (0.22 ± 0.07 ng/mL versus 0.12 ± 0.03 ng/mL, p <0.001), higher placental concentration of manganese {0.99 (0.38 – 1.78) *μ*g/g versus 0.42 ± 0.18 *μ*g/g, p <0.001}, iron (252.16 ± 170.61 *μ*g/g versus 78.45 ± 51.73 *μ*g/g, p <0.001), copper {2.96 ± 1.80 *μ*g/g versus 1.62 (0.70 – 3.88) *μ*g/g, p 0.019}, zinc {58.34 (27.88 – 124.05) *μ*g/g versus 28.41 (1.46 – 137.69) *μ*g/g, p 0.011}, selenium (0.31 ± 0.31 ng/g versus 0.14 ± 0.20 ng/g, p 0.024), AtRA {21.7 ± 10.69 ng/g versus 0.7 (0.42 – 5.10) ng/g, p <0.001}, and 25(OH)D {75.84 ± 45.12 ng/g versus 18.00 (5 – 88) ng/g, p <0.001}, lower placental concentration of mercury (0.20 ± 0.17 ng/g versus 20.47 ± 41.35 ng/g, p 0.019) and lead (0.02 ± 0.01 ng/g versus 0.81 ± 1.43 ng/g, p 0.009), and higher cord blood concentration of copper {32.20 (16.30 – 69.60) *μ*g/dL versus 20.60 (5.80 – 53.30) *μ*g/dL, p 0.006} and AtRA (0.16 ± 0.04 versus 0.07 ± 0.01, p <0.001).

**Conclusion:**

Preterm birth is associated with lower concentrations of micronutrients which play a role in antioxidant mechanism, as well as higher concentration of mercury and lead.

## 1. Introduction

Preterm birth, defined as babies born alive before 37 weeks of pregnancy, remains the leading cause of mortality and morbidity of children under the age of 5 in global scale, particularly in a developing country such as Indonesia. As much as 15 out of 100 babies are delivered prematurely, contributing to 35.5% of neonatal death in the country [1]. While its incidence contributes to almost 50% of birth in Cipto Mangunkusumo Hospital, Indonesia (2015–2017), the precise mechanism of preterm birth remains unclear. The cellular apoptosis that transmits an inflammatory signal, senescent placental cells, and its changes in placental membrane were hypothesized to stimulate parturition, both term and preterm birth, called as “common pathway.” The premature aging of the placenta caused by oxidative stress is believed to stimulate the common pathway prematurely. Reactive Oxygen Stress (ROS), the source of oxidative stress, activates NF-kappa B which further stimulates COX-2 and proinflammatory cytokines. Infection and exogenous factor such as lead exposure also upregulate ROS, increasing the risk of preterm birth. Micronutrients such as trace elements and vitamins such as copper, zinc, manganese, selenium, and vitamin A serve as endogenous antioxidant that counterbalances the oxidative stress, as well as regulating inflammatory response [2, 3].

Micronutrients are essential inorganic constituents for human health although only needed in minute quantities. Suboptimal status of micronutrients, whether deficiency or excess, could lead to detrimental pregnancy outcome, although it is difficult to identify since many pregnancy complications are often multifactorial. The knowledge of trace elements and vitamins role has been progressive for the past 40 years. Essential trace elements of the human body include iron (Fe), zinc (Zn), copper (Cu), selenium (Se), and manganese (Mn). These elements are related in so many enzymes that single trace element deficiency is often not associated with specific clinical manifestation, but it manifests as a combination of various symptoms [4, 5].

Studies have demonstrated the role of trace elements along with vitamin A and vitamin D in antioxidant activity which is very important in pregnancy. Preeclampsia, preterm birth, and intrauterine growth restriction are among pregnancy complications related to inflammatory state. Micronutrients also play role in the division and differentiation of fetal cells and their development [5].

Concentrations of micronutrients is affected by dietary habit, lifestyle, and environmental condition. Following industrial revolution, mercury (Hg) and lead (Pb) are gaining attention regarding environmental exposure and their effects in the body. During pregnancy, even low lead concentration could have adverse effects such as developmental delays, low birthweight, and miscarriage. Contamination of mercury occurs primarily through the consumption of contaminated seafood and rice grown in contaminated waters. It is actively transported across the placenta and impairs fetal neurodevelopment [6, 7].

We believe it is important to evaluate the status of micronutrients of pregnant women and whether its concentration is different between term and preterm birth in Indonesia. By gaining the knowledge about the exact role of micronutrients in the pregnancy, better nutrient approach of pregnancy would be achieved to optimize the normal pregnancy and to reduce the incidence of pregnancy complications, particularly preterm birth.

## 2. Methods

A cross-sectional study was conducted to expand the knowledge about the status of micronutrients in pregnant women who gave birth at term and preterm. The measured outcomes were maternal serum, placental, and blood cord concentration of zinc, copper, iron, selenium, manganese, mercury, lead, AtRA, and 25(OH)D. Subjects were selected by consecutive sampling technique.

Sample was taken in Cipto Mangunkusumo National Central General Hospital and Budi Kemuliaan Hospital during January–June 2017. This study has been approved by Ethics Committee of Faculty of Medicine of Universitas Indonesia (LB.01.01/X.2/179/2016).

The inclusion criteria were pregnant women undergoing preterm birth in 26–36 weeks of gestational age for the preterm groups and ≥37 weeks of gestational age for the term group and agreed to participate by signing informed consent. The exclusion criteria were subjects with multiple pregnancies, intrauterine growth restriction (IUGR), fetal congenital anomaly, preterm premature rupture of membrane (PPROM), and other comorbidities (hypertension in pregnancy, preeclampsia, gestational diabetes mellitus, heart disease, and autoimmune disease).

All of the samples were obtained soon after delivery. 15 cc of maternal blood sample was obtained from the vein and then put into serum separator tube (SST) for AtRA and 25(OH)D measurement as well as trace element clot activator tube to measure other trace elements. Samples were then sent within 30 minutes to Prodia Laboratory, Jakarta. Samples were centrifuged for 10 minutes and were kept at –80°C. Placental tissues were taken from marginal placenta and full-thickness-parenchymal placenta. Each sample was put into phosphate-buffered saline (PBS) solution and then kept at 4°C for maximum 24 hours.

Zinc, copper, iron, selenium, manganese, mercury, and lead were measured by Inductively Coupled Plasma-Mass Spectrometry (ICP-MS) using Agilent-MS 7700x series. Samples were diluted to 2 mL with a matrix solution containing 0.05 mL of concentrated ammonia, 1 mL of 0.01 M disodium ethylenediaminetetraacetate, 0.7 mL of Triton X-100, and 20 mL of butanol per liter [8]. AtRA and 25(OH)D were measured by Liquid Chromatography-tandem Mass Spectrometry (LC-MS/MS) technique with electrospray ionization following liquid-liquid extraction, solid-phase extraction, and derivatization with 4-phenyl-1,2,4-triazoline-3,5-dione (PTAD). The instruments used in the technique were Agilent Liquid Chromatography (LC) System 1290 with Agilent Triple Quad 6460 (LC/MS). In AtRA isomer separations, the retention times were set to 8.0 minutes for blood sample and 14.3 minutes for placenta. All mass transitions used dwell time of 50 ms and fragmentor voltage of 100V. Other parameters were as follows: electron multiplier voltage = 400V, nitrogen gas flow rate = 9 L/min, nebulizer pressure = 40 psi, sheath gas temperature = 250°C, sheath gas flow rate = 7 L/min, and capillary potential = 3000V [9, 10]. The examinations were performed in Prodia Laboratory, Jakarta.

Data was processed using IBM SPSS version 20 program. Data analysis was conducted using Shapiro-Wilk normality test, hypothesis test according to variable type and data normality, and correlation test according to data normality. The data were supplemented with 95% confidence interval, with the significance limit being set to p ≤ 0.05.

## 3. Results

**Figure 1:**
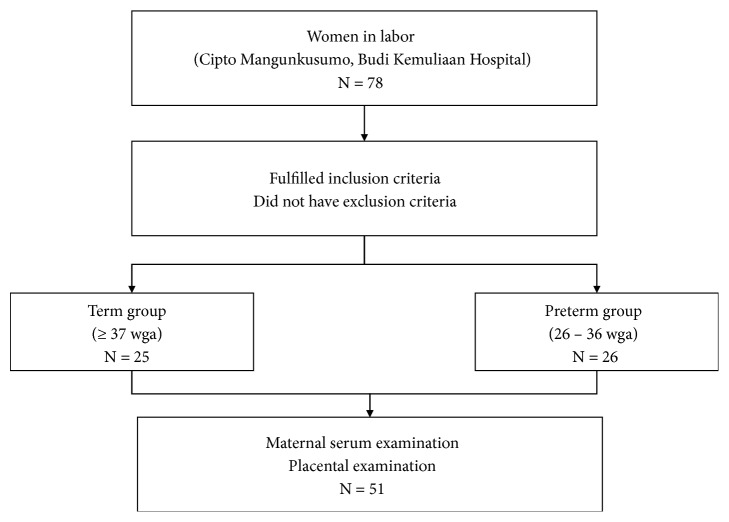
Participants flowchart.

A total of 51 pregnant women were selected, divided into term group and preterm group.  Table 1 presents characteristics of subjects. Participant flowchart is depicted in Figure 1. Body height of the preterm group was higher than the term group. Age and nutritional status depicted as body mass index (BMI) and upper arm circumference (UAC) were not different between groups.

Our finding about higher stature in preterm group contradicted with several studies that showed that lower maternal stature was associated with decreasing gestational age mainly due to maternal anatomical constraints and immature physical development in teenage pregnancy [11–13]. The different result might be due to other factors of preterm birth which dominated subjects such as infection and placental insufficiency.

Table 2 summarizes maternal obstetric status and the pregnancy outcome. The outcomes of preterm and term birth were obviously different. However, no difference of parity, preterm history, and birth method was observed.

Table 3 presents the concentration of micronutrients obtained from maternal serum, placenta, and cord blood. In maternal serum, only AtRA out of other elements was significantly higher in term group. Placental trace elements had more remarkable difference between groups. Term group had higher concentration of manganese, iron, copper, zinc, selenium, AtRA, and 25(OH)D, as well as lower concentration of mercury and lead compared to preterm group. Cord blood samples showed higher concentration of copper, selenium, and AtRA in term group.

Both groups had concentration of serum zinc and 25(OH)D below normal range as well as serum copper above normal range. The concentration of zinc and 25(OH)D corresponds to another study in Indonesia that as much as 81.2% of its population have zinc deficiency and 99.6% have 25(OH)D deficiency [14]. Currently there is no reference of normal value for placental and cord blood concentration of trace elements as well as serum AtRA concentration.

Term group had higher concentration of serum AtRA. AtRA is a vitamin A derivate that decreases cytokine expression in vitro and regulates Treg cells to promote anti-inflammation and differentiation of T effector cells. It also restricts NF-*κ*B activity through activation of retinoic acid receptor, resulting in reduction of inflammatory state. This mechanism might be the explanation of protective effects of AtRA in preventing preterm birth [15, 16].

The results of supplementation of vitamin A in pregnancy are contradictory. The reports, consisting of 35 trials, were summarized by a Cochrane report and were concluded that vitamin A supplementation during pregnancy does not help to prevent preterm birth directly, but it reduces maternal anemia and maternal infection. However, multiple studies in the report showed that vitamin A deficiency is associated with preterm birth. This might highlight the importance of reaching adequate preconceptional nutrition [17].

In contrast with serum findings, the trace elements in placenta were significantly different between two groups. Placental concentration of what is known to play anti-inflammatory function such as manganese, copper, zinc, and selenium was higher in term group. During placentation, the release of trophoblast plugs with flow of blood into the intervillous space leads to the generation of oxidative stress. However, placenta is armed with antioxidant including selenium-dependent enzymes of glutathione peroxidase, thioredoxin reductases, selenoprotein-P, as well as copper/zinc, and manganese superoxide dismutase, which require the mentioned trace elements [18]. The failure to keep the redox imbalance, i.e. pro-oxidant and anti-oxidant by these endogenous antioxidant systems lead to DNA damage and telomere shortening, accelerating telomere-dependent senescence of fetal membrane, causing senescence-associated inflammatory that lead to parturition. Several studies had demonstrated increased oxidized metabolites (malondialdehyde) and reduced concentration of antioxidant (GSH, selenium, and GSH-T) in preterm birth [3].

Some trace elements undergo active transport. Nandakumaran et al. reported that manganese is actively transported across the placental membrane, explaining the difference in manganese placental concentration despite no significant difference of manganese concentration in maternal serum [19]. Terrin et al. also stated that placental transfer of zinc to the fetus is also an active process, mediated by endocytic mechanism. Zinc deficiency in the fetus is observed only in the presence of severe maternal zinc deficiency, because the active transport maintained placental zinc concentration constantly higher than maternal levels [20].

Lower copper concentrations are observed in both placental and cord blood of preterm group. This is expected because fetal serum copper concentrations reach a maximum at the end of the last trimester of pregnancy, whereas the liver of premature infant is immature and cannot accumulate copper. Copper is bound to chaperone proteins, which deliver it to the target molecule. There are two Cu-ATPases expressed in placenta, ATP7A and ATP7B [21, 22]. Selenium transfer also primarily happens in the third trimester which accumulates in the fetal liver, explaining the lower selenium concentration in placental and cord blood of preterm group [23].

Although serum concentration was below normal range, placental concentration of 25(OH)D was also higher in term group compared to preterm group. Not only beneficial to bone metabolism, vitamin D along with its derivatives plays a role in immune system, particularly innate immune response. Deficiency in vitamin D is associated with poor pregnancy outcomes including intrauterine growth restriction and preterm birth [14].

Despite lower placental iron concentration in preterm birth, the iron cord blood concentration is not different between groups. The most circulating fetal iron will be metabolized by fetus leaving less iron in fetal circulation, which is interrupted by preterm birth, resulting in iron stores at birth being proportional to birthweight. The seemingly normal iron concentration in preterm cord blood might not indicate the real iron store but rather a systemic inflammatory response that is common in preterm infants. Placenta exhibits great capacity to mobilize iron for fetal use regardless of maternal status. Hepcidin, the iron regulatory “hormone” expressed by the liver, negatively regulates cellular iron transport via an FPN1-dependent mechanism, although several studies have contradictory results [24, 25].

On the other hand, preterm group showed higher placental concentrations of lead and mercury, but no significant difference was found in the cord blood sample. Lead readily crosses the placenta by passive diffusion and accumulates in placenta and fetal tissues, such as fetal liver. Depending on its form, mercury could cross the placenta by passive diffusion (vapor mercury) or active transport (methyl mercury). Metallothionein (MT) is likely responsible for binding the heavy metals in the placenta. There is evidence that mercury and lead are able to induce MT synthesis in placenta so that they do not cross the placenta to the fetal blood [26]. Mercury exposure is known to cause cell death by cytotoxicity and induction of apoptosis, as well as oxidative stress [27, 28]. Lead exposure is also known to promote apoptosis by hyperacetylation [29]. However, the present studies were not done in placental cells. In this study, cord blood lead was lower than serum in both groups; contrarily cord blood mercury was higher in cord blood than serum, which is in accordance with several studies [8, 30]. This might show another selective barrier role of placenta to lead but not to mercury. This study, however, did not identify the source of heavy metals exposure. As Cipto Mangunkusumo Hospital is a national referral hospital whose patients' backgrounds are very diverse, we hypothesized that socioeconomic status and environmental factor might lead to different placental concentration of lead and mercury.

We did not observe any correlation between maternal serum, placental, and cord blood of the same elements except AtRA that correlated positively between all the specimens {p <0.001, r 0.703 (placental and cord blood), r 0.570 (placental and maternal serum), and r 0.620 (maternal serum and cord blood)}. It was found, however, that many elements in placenta had negative correlation with mercury and lead and also positive correlation with each other.

Several studies showed that micronutrient supplementation and higher concentration of zinc and iron have a positive impact to mitigate lead toxicity [31, 32]. Pb had moderate positive correlation with Hg, which might be caused from contaminated environment. It is speculative to say that the micronutrients Mn, Fe, Cu, Zn, Se, AtRA, and 25(OH)D had synergistic mechanism to fight the “unwanted” ones, Hg and Pb. However, much is unknown regarding placenta and its homeostasis and protective mechanism.

## 4. Conclusion

The difference of micronutrients concentration was found between term and preterm births. Preterm birth is associated with lower concentrations of micronutrients which play role in antioxidant mechanism, as well as higher concentration of heavy metals such as mercury and lead. Compared to preterm birth group, term birth group had higher concentration of AtRA in maternal serum, higher placental concentration of manganese, iron, copper, zinc, selenium, AtRA, and 25(OH)D, lower placental concentration of mercury and lead, and higher concentration of copper and AtRA in cord blood.

## Figures and Tables

**Table 1:** Characteristics of subjects.

Variable	Term (n = 25)	Preterm (n=26)	p
Age (years)	27.68 (6.47)	24 (17 – 41)	0.463^a^
Body height (cm)	153.44 (5.79)	156.70 (4.81)	**0.034** ^**b**^
BMI (kg/m^2^)	21.75 (3.22)	22.17 (4.65)	0.707^b^
UAC			
< 23.5 cm	6	9	0.127^c^
≥23.5 cm	19	17	

BMI: body mass index, UAC: upper arm circumference.

^a^Mann-Whitney test. ^b^Independent *t*-test. ^c^Chi-square test.

**Table 2:** Maternal obstetric status and pregnancy outcome. Apart from the apparent characteristic of preterm birth, no significant difference was found between groups.

**Variable**	**Term**	**Preterm**	**P**
	**(n = 25)**	**(n = 26)**	
Parity			
Nulliparous	18	17	0.754^a^
Multiparous	7	10	
History of preterm			
No	22	23	0.959^a^
Yes	3	3	
Gestational age upon birth (weeks)	39 (37–41)	33 (26–36)	**<0.001** ^**b**^
Birth method			0.938^a^
Spontaneous	19	21	
Caesarian section	6	6	
**Infant data**			
Birth weight (g)	3056.2 (335.32)	1897.88 (549.023)	**<0.001** ^**c**^
Body length (cm)	49.02 (2.58)	42.92 (5.12)	**<0.001** ^**c**^
Abdominal circumference (cm)	30.88 (2.03)	27.04 (3.58)	**<0.001** ^**c**^
Head circumference (cm)	32.42 (1.54)	29.13 (2.64)	**<0.001** ^**c**^
Placental weight (g)	554.8 (92.6)	426.77 (102.46)	**<0.001** ^**c**^

^a^Chi-square test. ^b^Mann-Whitney test. ^c^Independent *t*-test.

**Table 3:** Trace elements concentration of maternal serum, placenta, and cord blood. Placental concentration of all trace elements shows higher antioxidant concentration and lower concentration of mercury and lead, while only AtRA shows significant difference in maternal serum, as well as copper, selenium, and AtRA in cord blood.

**Variable**	**Normal**	**Term (n = 25)**	**Preterm (n = 26)**	**P**
**Maternal serum**				
Manganese (*μ*g/L)	≤ 1.1	1.00 (0.50 – 1.70)	1.09 (0.64)	0.428^a^
Iron (*μ*g/dL)	35 – 145	77.00 (24.00 – 221.00)	71.50 (22.00 – 234.00)	0.977^a^
Copper (*μ*g/dL)	75 – 145	222.65 (0 – 376.80)	215.35 (68.40 – 313.70)	0.655^a^
Zinc (*μ*g/dL)	60 – 130	45.16 (9.32)	40.26 (13.96)	0.116^b^
Mercury (*μ*g/L)	≤ 9	2.33 (1.49)	3.14 (2.19)	0.178^b^
Lead (*μ*g/dL)	≤ 9	3.25 (1.50 – 7.60)	2.81 (1.21)	0.177^a^
Selenium (*μ*g/L)	23 - 190	76.42 (16.30)	72.77 (18.04)	0.458^b^
AtRA (ng/mL)		0.22 (0.07)	0.12 (0.03)	**<0.001** ^**b**^
25(OH)D (ng/mL)	30 - 44	15.42 (5.67)	14.00 (3 – 53)	0.760^a^

**Placenta**				
Manganese (*μ*g/g)		0.99 (0.38 – 1.78)	0.42 (0.18)	**<0.001** ^**a**^
Iron (*μ*g/g)		252.16 (170.61)	78.45 (51.73)	**<0.001** ^b^
Copper (*μ*g/g)		2.96 (1.80)	1.62 (0.70 – 3.88)	**0.019** ^**a**^
Zinc (*μ*g/g)		58.34 (27.88 – 124.05)	28.41 (1.46 – 137.69)	**0.011** ^**a**^
Mercury (ng/g)		0.20 (0.17)	20.47 (41.35)	**0.019** ^**b**^
Lead (ng/g)		0.02 (0.01)	0.81 (1.43)	**0.009** ^**b**^
Selenium (ng/g)		0.31 (0.31)	0.14 (0.20)	**0.024** ^**b**^
AtRA (ng/g)		21.7 (10.69)	0.7 (0.42 – 5.10)	**<0.001** ^**a**^
25(OH)D (ng/g)		75.84 (45.12)	18.00 (5 – 88)	**<0.001** ^**a**^

**Cord blood**				
Manganese (*μ*g/L)		3.40 (2.10 – 19.40)	3.19 (1.08)	0.327^a^
Iron (*μ*g/dL)		212.00 (111.00 – 1324.00)	236.50 (131.00 – 803.00)	0.380^a^
Copper (*μ*g/dL)		32.20 (16.30 – 69.60)	20.60 (5.80 – 53.30)	**0.006** ^**a**^
Zinc (*μ*g/dL)		293.80 (234.59)	321.43 (176.59)	0.210^b^
Mercury (*μ*g/L)		3.50 (0.90 – 12.00)	4.63 (2.54)	0.461^a^
Lead (*μ*g/dL)		2.35 (0.80 – 5.70)	1.90 (0.70 – 3.80)	0.244^a^
Selenium (*μ*g/L)		49.65 (12.71)	41.83 (9.48)	**0.021** ^**b**^
AtRA (ng/mL)		0.16 (0.04)	0.07 (0.01)	**<0.001** ^**b**^
25(OH)D (ng/mL)		13.10 (6.25)	12.56 (5.17)	0.731^b^

^a^Mann-Whitney test. ^b^Independent *t*-test.

## Data Availability

The data used to support the findings of this study are available from the corresponding author upon request.
